# Supplementary material for: L-Dopa-Decarboxylase (DDC) Is a Positive Prognosticator for Breast Cancer Patients and Epinephrine Regulates Breast Cancer Cell (MCF7 and T47D) Growth In Vitro According to Their Different Expression of Gi- Protein- Coupled Receptors
Source: Int J Mol Sci. 2020 Dec 15;21(24):9565. doi: 10.3390/ijms21249565 (PMC7765554; doi:10.3390/ijms21249565)
Supplement: Supplementary file 1 [file ijms-21-09565-s001.pdf]

patient no.	T	N	M	G	Her 2	ER	PR	Luminal	TAAR1 (nuclear)		DDC (cytoplasma)	
									Intensity	IRS	Intensity	IRS
1	1b	x	x	3	pos	pos	neg	B	0	0	2	8
2	1c	0	x	2	neg	pos	pos	A	0	0	2	8
3	1b	0	x	2	neg	pos	pos	B	0	0	n.a.	n.a.
4	1b	1bi	x	2	neg	neg	neg		0	0	n.a.	n.a.
5	1c	1biii	x	2	neg	pos	neg	B	0	0	3	12
6	2	2	x	3	neg	pos	pos	A	2	6	n.a.	n.a.
7	2	1bii	x	2	neg	pos	pos	B	0	0	3	12
8	1c	1biv	x	3	neg	pos	pos	A	0	0	1	4
9	2	1bii	x	2	neg	pos	pos	A	0	0	n.a.	n.a.
10	1c	1biii	x	3	neg	pos	neg		0	0	3	12
11	1c	0	x	2	neg	pos	pos	A	0	0	n.a.	n.a.
12	1c	1bi	x	3	neg	pos	pos	B	0	0	1	4
13	1b	1bi	x	3	pos	pos	pos		3	9	n.a.	n.a.
14	1c	0	x	2	neg	pos	pos	B	0	0	2	8
15	1c	1bi	x		neg	pos	pos	A	0	0	2	8
16	1c	0	x	3	neg	pos	pos	A	0	0	2	8
17	1c	x	x	2	neg	pos	pos	A	0	0	n.a.	n.a.
18	1c	0	x	2	neg	pos	pos	A	0	0	1	4
19	1c	0	x	2	neg	pos	neg	A	0	0	3	12
20	2	1a	x	2	neg	pos	pos	A	2	2	2	8
21	1b	0	x	2	neg	neg	neg		2	4	1	4
22	1c	0	x		neg	neg	neg		2	4	1	4
23	2	1biii	x	3	neg	pos	neg	A	2	4	2	8
24	1c	1biii	x	2	neg	pos	pos	A	0	0	n.a.	n.a.
25	1c	1a	x	3	neg	pos	neg	A	0	0	2	8
26	1c	0	x	2	neg	pos	pos	A	0	0	2	8
27	1c	1a	x	2	pos	pos	pos		0	0	3	12
28	1c	1bi	x	2	neg	pos	neg	B	0	0	1	4
29	1c	1bi	x	3	neg	pos	pos	A	0	0	1	3
30	1c	o	x	3	neg	neg	pos	B	2	2	1	4
31	2	x	x	2	neg	pos	pos	A	0	0	0	0
32	2	x	x	2	neg	pos	neg	A	0	0	1	4
33	1c	0	x	2	neg	pos	pos	A	0	0	1	4
34	1b	0	x	3	neg	pos	pos	B	1	1	2	8
35	2	1bi	x	3	neg	pos	pos	A	1	1	n.a.	n.a.
36	1c	0	x	2	neg	pos	neg	A	0	0	2	4
37	2	0	x	2	neg	neg	neg		0	0	2	8
38	2	0	x		neg	pos	neg	A	0	0	0	0
39	2	0	x	3	neg	pos	pos	B	0	0	1	4
40	1b	0	x	2	neg	pos	pos	B	0	0	2	8
41	2	1biii	x	3	pos	neg	neg		0	0	2	8
42	2	0	x	3	pos	pos	pos		0	0	3	12
43	1c	1bi	x	3	neg	pos	pos	A	0	0	n.a.	n.a.
44	1b	0	x		neg	pos	pos	A	0	0	0	0
45	2	1biv	x	3	pos	pos	pos		0	0	2	8
46	1c	0	x	3	neg	neg	neg		0	0	2	8
47	1c	x	x	2	neg	pos	pos	A	0	0	1	4
48	1c	0	x	2	neg	neg	neg		0	0	3	12
49	2	0	x	2	neg	pos	pos	A	0	0	2	8
50	1c	0	x	3	neg	pos	pos	A	0	0	3	12
51	2	x	x		neg	pos	neg	A	0	0	3	12
52	2	0	x		neg	pos	pos	A	0	0	3	12
53	1c	1bi	x	3	pos	pos	pos		0	0	3	12
54	1c	0	x	3	pos	pos	neg		0	0	0	0
55	2	1biii	x	3	neg	neg	neg		0	0	1	4
56	2	1biv	x	3	pos	pos	neg		0	0	2	8
57	1c	0	x	3	neg	pos	pos	A	0	0	2	8
58	1b	1biii	x	3	neg	neg	neg		0	0	1	4
59	2	1biii	x	2	neg	pos	pos	B	3	12	1	4
60	1b	1biii	x	2	neg	pos	neg	A	3	3	2	8
61	1c	0	x	2	neg	pos	neg	B	0	0	2	8
62	2	0	x		neg	neg	neg		0	0	1	3
63	1c	0	x	2	neg	pos	pos	B	3	3	1	3
64	4a	1biii	x		neg	pos	neg	A	2	6	0	0
65	1c	0	x	2	neg	pos	pos	A	0	0	1	4
66	1c	0	x		neg	pos	pos	B	0	0	2	8
67	1c	0	x	2	neg	pos	pos	B	0	0	3	12
68	1b	0	x	2	neg	pos	pos	A	3	6	1	4
69	1c	0	x	3	neg	pos	neg	A	0	0	1	4
70	2	1biii	x	3	neg	neg	neg		3	3	1	3
71	2	0	x	3	neg	neg	neg		0	0	2	8
72	2	0	x	3	neg	pos	pos	A	3	9	1	3
73	1b	0	x	2	neg	pos	neg	B	0	0	2	6
74	1c	1bii	x	3	neg	pos	pos	A	0	0	2	6
75	1c	1biii	x	2	neg	pos	pos	A	0	0	1	4
76	1c	1biii	x		pos	neg	neg		0	0	2	8
77	2	1bi	x	3	neg	pos	pos	A	0	0	1	4
78	2	1biii	x	2	neg	pos	neg	A	0	0	1	4
79	2	1bi	x	3	neg	neg	neg		0	0	1	4
80	1c	0	x		neg	pos	pos	A	0	0	0	0
81	2	1biii	x	3	neg	pos	pos	B	0	0	3	12
82	1c	1biii	x		neg	pos	neg	B	0	0	n.a.	n.a.
83	1c	1bi	x	3	neg	neg	neg		0	0	0	0
84	4b	2	1	3	neg	neg	neg		0	0	0	0
85	1b	1bi	x	3	neg	pos	pos	B	0	0	3	12
86	1b	0	x		neg	pos	pos	A	0	0	2	8
87	1b	1biii	x	3	neg	pos	pos	B	0	0	1	4
88	2	0	x	3	neg	neg	neg		0	0	2	8
89	2	1bi	x	2	neg	pos	pos	B	0	0	2	8
90	2	2	x	2	neg	pos	pos	A	0	0	2	4
91	2	0	x	2	neg	pos	pos	B	0	0	2	8
92	1c	1biii	x	2	neg	pos	pos	A	0	0	3	12
93	2	1bi	x	2	neg	pos	pos	A	0	0	2	8
94	2	0	x	2	neg	neg	pos	B	0	0	0	0
95	2	2	x	3	neg	pos	pos	A	0	0	0	0
96	1c	1biii	x	2	neg	neg	neg		3	3	0	0
97	4d	1biii	x		pos	pos	pos		0	0	0	0
98	1c	x	x	2	neg	pos	pos	B	0	0	0	0
99	1c	0	x	3	neg	pos	pos	B	0	0	1	3
100	4b	1biv	x	2	neg	pos	pos	B	0	0	2	8
101	1c	0	x	2	neg	pos	pos	B	3	6	1	3
102	1c	0	x	3	neg	neg	pos	B	0	0	0	0
103	1b	0	x		pos	neg	neg		0	0	2	8
104	2	1biii	x		neg	pos	neg	A	0	0	1	2
105	2	1bi	x	3	neg	neg	neg		0	0	0	0
106	1c	1bi	x		neg	pos	pos	B	0	0	2	6
107	3	1biii	x	3	neg	pos	neg	B	3	6	0	0
108	1b	1biii	x	2	neg	pos	neg	A	0	0	1	3
109	1c	1bi	x		neg	pos	pos	B	2	4	2	8
110	2	1biii	x	3	neg	neg	neg		2	4	3	12
111	1c	0	x		neg	pos	neg	A	0	0	2	8
112	1c	2	x	3	pos	neg	neg		2	4	3	12
113	1c	1a	x	2	pos	pos	neg		0	0	1	3
114	1c	1bi	x	2	neg	pos	pos	A	0	0	3	12
115	1c	0	x	2	neg	pos	pos	A	0	0	0	0
116	1c	0	x	2	neg	pos	pos	A	0	0	0	0
117	1c	0	x	1	neg	pos	pos	A	0	0	2	4
118	1c	0	x	1	neg	pos	neg	A	0	0	1	4
119	1b	0	x	1	neg	pos	neg	A	0	0	1	3
120	1c	0	x		neg	neg	neg		0	0	2	8
121	1b	0	x		neg	pos	pos	A	0	0	2	8
122	2	0	x		neg	pos	neg	B	0	0	3	12
123	1c	2	x		neg	pos	pos	A	0	0	3	12
124	1b	0	x	2	neg	neg	neg		0	0	2	8
125	1c	0	x	2	neg	pos	neg	A	0	0	3	12
126	1c	1biii	x		pos	pos	pos		0	0	2	8
127	2	1bii	x		neg	pos	pos	A	0	0	3	12
128	1c	x	x	1	neg	pos	pos	A	0	0	1	4
129	2	0	x		neg	pos	pos	B	0	0	1	2
130	1b	0	x		neg	pos	pos	A	0	0	2	8
131	1b	0	x		neg	pos	pos	B	0	0	0	0
132	1b	0	x		neg	pos	pos	A	0	0	3	12
133	1b	1a	x		neg	pos	pos	A	0	0	0	0
134	1b	0	x		neg	pos	pos	A	0	0	1	4
135	1c	1biv	x		neg	neg	neg		0	0	3	12
136	1c	1biii	x		neg	pos	pos	A	0	0	3	12
137	1c	0	x	1	neg	pos	pos	A	0	0	1	4
138	1b	1b	x		neg	pos	pos	A	0	0	3	12
139	1c	0	x		neg	pos	pos	A	0	0	3	12
140	1c	1biii	x	2	neg	pos	pos	B	0	0	2	6
141	1c	0	x	2	neg	pos	pos	A	0	0	1	3
142	1c	0	x		neg	pos	pos		0	0	2	4
143	1c	0	x	2	neg	pos	pos	B	0	0	0	0
144	1b	0	x	2	neg	pos	pos	B	0	0	1	4
145	1c	0	1		neg	pos	pos	B	0	0	3	12
146	2	x	x	2	neg	neg	neg		0	0	2	2
147	1c	1biv	x		neg	pos	pos	A	0	0	1	3
148	1c	0	x	1	pos	pos	neg		0	0	0	0
149	2	1bii	x	2	pos	pos	pos		0	0	1	3
150	1c	0	x	2	neg	neg	neg		0	0	1	3
151	2	x	x	1	neg	pos	pos	A	0	0	1	4
152	1c	1bi	x	1	neg	pos	neg	B	0	0	0	0
153	2	1biv	x		pos	pos	neg		0	0	0	0
154	1c	0	x	1	neg	pos	neg	B	0	0	0	0
155	1c	0	x		neg	pos	pos	A	0	0	2	6
156	1b	1bi	x	3	pos	neg	neg		0	0	2	6
157	1c	0	x		neg	pos	pos	A	0	0	1	4
158	2	0	x		neg	pos	pos	B	0	0	2	8
159	1c	0	x	1	neg	pos	pos	B	3	3	2	8
160	2	1biii	x	3	neg	pos	neg	B	0	0	1	4
161	1c	0	x	1	neg	pos	pos	B	0	0	2	8
162	2	1biv	x	2	neg	pos	neg	B	0	0	2	8
163	1b	0	x	2	neg	pos	pos	B	0	0	1	4
164	2	1biii	x		neg	pos	pos	B	3	3	1	4
165	1b	0	x		neg	pos	pos		0	0	2	6
166	1c	2	x	2	neg	pos	neg	B	0	0	2	8
167	1b	0	x		neg	pos	pos	A	0	0	1	4
168	1c	0	x		neg	pos	pos	A	0	0	2	8
169	1b	1bi	x	1	neg	pos	neg	B	0	0	1	4
170	1b	0	x		neg	neg	neg		0	0	2	8
171	1c	0	x	1	neg	pos	pos	A	0	0	1	4
172	1c	0	x	2	neg	pos	pos	A	0	0	2	8
173	1b	1bi	x	2	neg	pos	neg	B	3	3	n.a.	n.a.
174	1c	0	x		neg	pos	neg	B	0	0	3	12
175	is				pos	pos	neg		0	0	2	8
176	1c	0	x	2	neg	pos	neg	B	0	0	3	12
177	1c	1biii	x		neg	pos	pos	A	0	0	2	8
178	2	2	x		neg	pos	pos	A	0	0	3	12
179	1c	1biv	x	2	pos	pos	neg		3	3	2	8
180	1	0	x	3	neg	neg	neg		0	0	1	4
181	1b	x	x		pos	pos	pos		3	3	2	6
182	1b	0	x	2	neg	pos	pos	A	3	3	1	4
183	1b	0	x	2	FISH	pos	pos		0	0	3	12
184	1c	0	x	3	neg	pos	neg	B	0	0	1	4
185	1c	0	x	1	neg	pos	pos	A	3	3	1	3
186	1b	0	x		neg	pos	pos	A	3	3	0	0
187	1c	0	x	2	neg	pos	pos	B	3	3	1	4
188	1b	0	x		neg	neg	neg		3	3	2	8
189	1b	0	x	2	neg	neg	neg		3	3	2	8
190	1b	0	x	2	neg	pos	pos	B	3	3	2	8
191	1c	0	x	3	neg	pos	pos	B	3	6	1	4
192	2	1biii	x		neg	pos	pos	B	3	3	3	9
193	1c	1biii	x		neg	pos	pos	A	3	3	2	8
194	1b	0	x	2	neg	pos	pos	B	3	3	0	0
195	2	1biii	x	2	neg	pos	pos	A	0	0	2	8
196	2	2	x		neg	pos	neg	B	0	0	1	4
197	1b	0	x		neg	pos	pos	B	0	0	3	12
198	1c	0	x	3	neg	neg	neg		3	6	1	4
199	2	1biii	x	2	pos	pos	neg		2	2	2	8
200	2	0	x		neg	pos	neg	B	3	6	1	4
201	1c	0	x		neg	pos	pos	B	3	3	1	3
202	2	0	x	3	neg	pos	pos	B	0	0	1	4
203	2	0	x		neg	pos	pos	B	0	0	1	4
204	2	0	x	2	neg	pos	pos	B	0	0	1	4
205	1c	0	x	2	neg	pos	pos	A	3	3	1	4
206	1c	0	x		neg	neg	pos	B	0	0	0	0
207	2	1biii	x		neg	pos	pos	A	3	3	1	3
208	1b	0	x	2	neg	pos	pos	A	3	6	0	0
209	2	1biii	x		neg	pos	neg	B	3	6	0	0
210	1c	0	x		neg	pos	neg	A	0	0	3	12
211	1b	0	x	1	neg	pos	neg	A	0	0	0	0
212	2	0	x	3	pos	neg	neg		3	6	1	4
213	1c	0	x	2	neg	pos	neg	B	3	3	2	8
214	2	0	x	3	neg	neg	neg		3	9	2	8
215	1b	0	x	1	neg	pos	pos	A	3	9	n.a.	n.a.
216	2	2	x		neg	pos	neg	A	3	9	0	0
217	1b	0	x	2	neg	pos	neg	B	3	9	1	4
218	1c	0	x		pos	pos	pos		3	6	1	3
219	1b	0	x	2	neg	pos	pos	A	3	9	n.a.	n.a.
220	1c	1b	x	2	neg	pos	pos	A	3	6	2	8
221	1c	0	x		neg	pos	pos	A	3	6	0	0
222	4d	1bi	x		neg	neg	pos	A	3	3	1	4
223	1b	0	x		neg	pos	pos	A	3	6	2	8
224	2	1biii	x	2	neg	pos	neg	A	0	0	1	4
225	1b	0	x		neg	pos	pos	A	3	6	1	3
226	2	0	x	2	neg	pos	neg	B	3	3	0	0
227	1c	0	x		neg	pos	pos	B	3	12	2	8
228	1c	1bi	x		neg	pos	pos	A	0	0	3	12
229	1c	0	x		neg	neg	neg		3	3	1	4
230	2	1bi	x		neg	pos	neg	A	0	0	0	0
231	1c	0	x	3	neg	neg	neg		0	0	2	8
232	1b	0	x		neg	pos	pos	B	3	6	0	0
233	2	1bi	x	3	pos	neg	neg		3	6	1	4
234	2	1bi	x		neg	pos	neg	A	0	0	0	0
235	1c	0	x		neg	pos	pos	A	3	9	2	8

Table S1. Patients characteristics and immunohistochemistry staining results of nuclear TAAR1 and cytoplasmatic DDC. (TNM-classification, G- Grading, HER2-status, ER- estrogen receptor, PR- progesterone receptor, luminal classification, immunohistochemistry staining: nuclear TAAR1 (intensity and IRS), cytoplasmatic DDC (intensity and IRS))

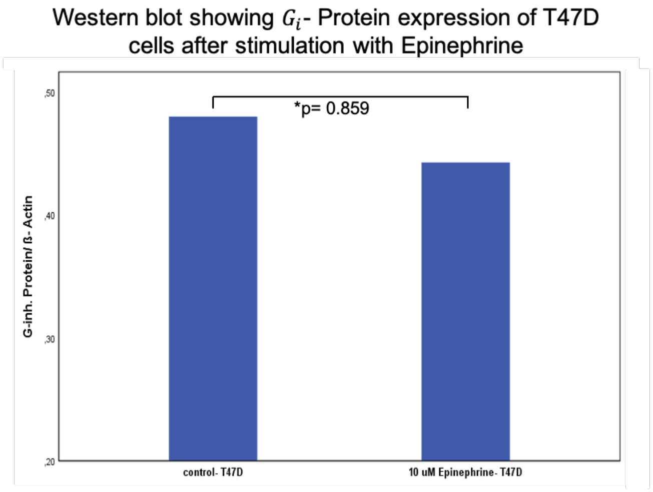

Figure S1. Western Blot analysis of G_i- protein expression in T47D cells after stimulation with Epinephrine. Bar chart of G_i- protein expression in T47D cells after incubation with 10μM Epinephrine. Epinephrine showed no significant impact on T47D (p= 0.859).
